# Local staging of ipsilateral breast tumor recurrence: mammography, ultrasound, or MRI?

**DOI:** 10.1007/s10549-020-05850-9

**Published:** 2020-08-08

**Authors:** Coco J. E. F. Walstra, Robert-Jan Schipper, Gonneke A. Winter-Warnars, Claudette E. Loo, Adri C. Voogd, Marie-Jeanne T. F. D. Vrancken Peeters, Grard A. P. Nieuwenhuijzen, Regina G. H. Beets-Tan

**Affiliations:** 1 Department of Surgery, Catharina Hospital Eindhoven, Michelangelolaan 2, 5623 EJ Eindhoven, The Netherlands; 2 Department of Radiology, The Netherlands Cancer Institute, Amsterdam, The Netherlands; 3 Department of Epidemiology, Maastricht University Medical Center, Maastricht, The Netherlands; 4 Department of Surgical Oncology, The Netherlands Cancer Institute, Amsterdam, The Netherlands; 5 GROW School for Oncology and Developmental Biology, Maastricht University Medical Centre, Maastricht, The Netherlands; 6 Department of Research, Netherlands Comprehensive Cancer Organization, Utrecht, The Netherlands

**Keywords:** Ipsilateral breast cancer recurrence, Tumor size estimation, Mammography, Breast ultrasound, Breast MRI

## Abstract

**Background:**

Despite increasingly effective curative breast-conserving treatment (BCT) regimens for primary breast cancer, patients remain at risk for an ipsilateral breast tumor recurrence (IBTR). With increasing interest for repeat BCT in selected patients with IBTR, a reliable assessment of the size of IBTR is important for surgical planning.

**Aim:**

The primary aim of this study is to establish the performance in size estimation of XMG, US, and breast MRI in patients with IBTR. The secondary aim is to compare the detection of multifocality and contralateral lesions between XMG and MRI.

**Patients and methods:**

The sizes of IBTR on mammography (XMG), ultrasound (US), and magnetic resonance imaging (MRI) in 159 patients were compared to the sizes at final histopathology. The accuracy of the size estimates was addressed using Pearson’s coefficient and Bland–Altman plots. Secondary outcomes were the detection of multifocality and contralateral lesions between XMG and MRI.

**Results:**

Both XMG and US significantly underestimated the tumor size by 3.5 and 4.8 mm, respectively, while MRI provided accurate tumor size estimation with a mean underestimation of 1.1 mm. The sensitivity for the detection of multifocality was significantly higher for MRI compared to XMG (25.5% vs. 5.5%). A contralateral malignancy was found in 4.4% of patients, and in 1.9%, it was detected by MRI only.

**Conclusion:**

The addition of breast MRI to XMG and US in the preoperative workup of IBTR allows for more accurate size estimation. MRI provides a higher sensitivity for the detection of multifocality compared to XMG.

**Electronic supplementary material:**

The online version of this article (10.1007/s10549-020-05850-9) contains supplementary material, which is available to authorized users.

## Introduction

Despite increasingly effective curative treatments for patients with primary breast cancer, women remain at risk for ipsilateral breast tumor recurrence (IBTR), with a cumulative risk of approximately 15% within 20 years [1]. As the prognosis of early recurrences detected by physical examination or screening mammography in asymptomatic patients is better than for patient-reported recurrences [2], international guidelines recommend annual follow-up after breast-conserving therapy (BCT). Follow-up consists of physical examination and mammography (XMG) and, when indicated, target ultrasonography (US) [3, 4]. In some cases, e.g., genetically predisposed patients or occult primary tumors on XMG and/or US, breast magnetic resonance imaging (MRI) is the technique of choice in follow-up imaging in the Netherlands [5].

In daily practice, breast MRI is frequently used in the workup for primary breast cancer patients to assess tumor size and multifocality, as this provides important information for eligibility for BCT and surgical planning. Evidence suggests that MRI is superior to XMG and US with respect to determining tumor size [6].

Furthermore, MRI has a higher sensitivity in comparison to XMG and US for the detection of multifocal lesions in primary breast cancer, i.e., the presence of two or more separate tumor foci in the breast, but it is at the expense of a higher false-positive rate [10]. As a result, more biopsies are being performed when MRI is added to XMG and US [11]. The presence of multifocality is associated with a higher local recurrence rate and worse overall survival [12]. The finding of additional tumor foci often results in alteration of surgical management, either by planning a wider excision or opting for mastectomy, as a lumpectomy in case of multifocality is regarded oncologically inferior [13, 14]. However, there is also evidence that breast-conserving surgery can be a safe treatment option even for multifocal tumors [15, 16]. Thus, multiple studies showed that while the addition of MRI in the workup of primary breast cancer led to a higher mastectomy rate, this had no effect on prognosis [7], except for patients with invasive lobular carcinoma (ILC) [10]. Hence, the oncological benefit of MRI in primary breast cancer is unclear.

Another important aspect of breast MRI is the incidental finding of clinically and mammographically occult malignancies in the contralateral breast. Like in detecting additional ipsilateral lesions, here too, the false-positive rate is high [17]. The incidence of MRI only (occult on conventional imaging) detected true contralateral malignancies is approximately 4% in patients with primary breast cancer [17]. After primary breast cancer treatment, patients have a 1.5–2 times higher relative risk to develop contralateral breast cancer compared to the general population [18]. Factors increasing this risk are young age at primary breast cancer [19], omission of endocrine or chemotherapy in primary breast cancer treatment [20], and carrying a BRCA-1/2 mutation [21]. The incidence of metachronous contralateral breast cancer is decreasing, most probably due to the increased use of adjuvant systemic therapy in primary breast cancer treatment [22, 23].

Currently, the surgical standard of care for ipsilateral breast tumor recurrences (IBTR) after BCT is salvage mastectomy. However, there is increasing evidence for the safety of repeat breast-conserving treatment (BCT) in selected cases of IBTR [24]. As large tumors and multifocality have been suggested contraindications for repeat BCT [25–27], it is important to obtain a reliable preoperative size estimation and to ensure the absence of additional tumor foci in the ipsilateral breast. This raises the question whether or not breast MRI should be part of the workup in patients with a ipsilateral breast tumor recurrence.

To date, there is no literature on the additional value of breast MRI in the diagnostic work up of patients with IBTR regarding size estimation and the detection of additional ipsilateral and contralateral tumor lesions. The primary aim of this study is to establish the performance in size estimation of XMG, US, and breast MRI in patients with IBTR. The secondary aim is to compare the detection of multifocality and contralateral lesions between XMG and MRI.

## Materials and methods

### Patient selection and data collection

In this single-center cohort study, the charts of 213 consecutive patients with IBTR diagnosed from January 2009 through December 2017 were retrospectively reviewed for eligibility. Inclusion criteria were:

1. Primary breast-conserving therapy with or without radiotherapy for invasive or in situ carcinoma
2. Histologically proven IBTR (invasive or carcinoma in situ)
3. Salvage IBTR surgery (mastectomy or repeat BCS); with or without systemic treatment prior to IBTR surgery
4. Availability of the imaging reports and histopathology reports of IBTR in the electronic patient file
5. Imaging of IBTR had to consist of either XMG, US or MRI or any combination of the three techniques

Exclusion criteria were chest wall recurrences following primary mastectomy, synchronous distant metastases treated with palliative intent and unavailability of imaging and/or histopathology reports.

### Outcomes

The primary aim of this study is to establish the performance in size estimation of XMG, US, and breast MRI in patients with IBTR compared with the findings in histopathology (including eventual extensive carcinoma in situ) of the surgical specimen, and for patients undergoing salvage mastectomy (2) the suspicion of multifocality on XMG and/or MRI and the finding of additional lesions in the ablative specimen.

A secondary outcome was the incidental finding of a contralateral tumor. As we could not establish the number of USs that were performed as a target US after XMG and/or MRI, we did not assess the value of US for contralateral lesion detection, but only for XMG and MRI.

The histopathology provided by either biopsy of the contralateral breast or contralateral ablative specimen was set as a reference for the presence of contralateral breast cancer.

In case of neoadjuvant systemic therapy (NST), either endocrine or chemo(-immuno)therapy, the tumor size measured on evaluation imaging after completion of NST was used for comparison to final histopathology of the surgical specimen. When there was no imaging after completion or premature termination of NST (or in case of endocrine therapy, more than four weeks between the last imaging and surgery), the case was excluded from the size estimation analysis. If a tumor was not visible on either XMG, US or MRI, they were still included in the size estimation analysis. These occult tumors were included with an estimated size set at 0 mm. To assess the effect of NST on the accuracy of the size assessment by MRI, analyses were calculated for both groups separately.

To calculate the additional value of MRI for multifocality assessment and the detection of contralateral findings, a comparison was made between the sensitivity of XMG and MRI.

### Imaging techniques

All mammograms were performed according to a standard protocol, which includes a mediolateral oblique and craniocaudal view of each breast. All mammograms were viewed by a dedicated breast radiologist, who also performed the target ultrasound of the breast. MRI technique and protocols were described previously [28]. In brief, breast MRI was performed using a 3.0 T Achieva scanner with a dedicated 7-element sense breast coil (Philips Medical Systems, Best, The Netherlands). In patients treated with NST, radiologically complete response was defined as the absence of pathologic (i.e., non-physiological) contrast enhancement in the original tumor region. For this study, radiological reports were checked and data of interest were obtained, in order to reflect the diagnostic performance of all three modalities in current daily practice.

### Statistical analysis

The correlation between measurements of the tumor size for each of the three imaging techniques and histopathology was calculated using Pearson’s correlation coefficient. Differences between the measurements of each imaging technique versus the histopathological findings were calculated per case using the formula “size at histopathology minus size on imaging”, compared using the student’s independent samples T-test and visualized using Bland–Altman plots [29]. As mentioned by Mann et al. and Lobbes et al., in their discussion on the best statistical method to present data regarding agreement between measurement modalities [35, 36], a good correlation does not automatically imply a good agreement between variables, as consistent overestimation and underestimation of the actual tumor size could lead to a good correlation coefficient, when the agreement is actually poor. To provide the most complete overview of the accuracy of size estimation by each imaging technique, we chose to use both techniques.

The detection of additional tumor foci and, where possible, contralateral malignancies was expressed in terms of sensitivity, specificity, positive predictive value (PPV), and negative predictive value (NPV) for each imaging technique. Differences in these outcomes were compared between imaging techniques using McNemar's symmetry X^2^-test. A size discrepancy between imaging and histopathology of ≥ 10 mm was considered clinically relevant, in line with other literature on this subject [30, 31]. All statistical analyses were performed using SPSS version 24 (SPSS Inc, Chicago, IL, USA).

## Results

### Inclusion

**Fig. 1:**
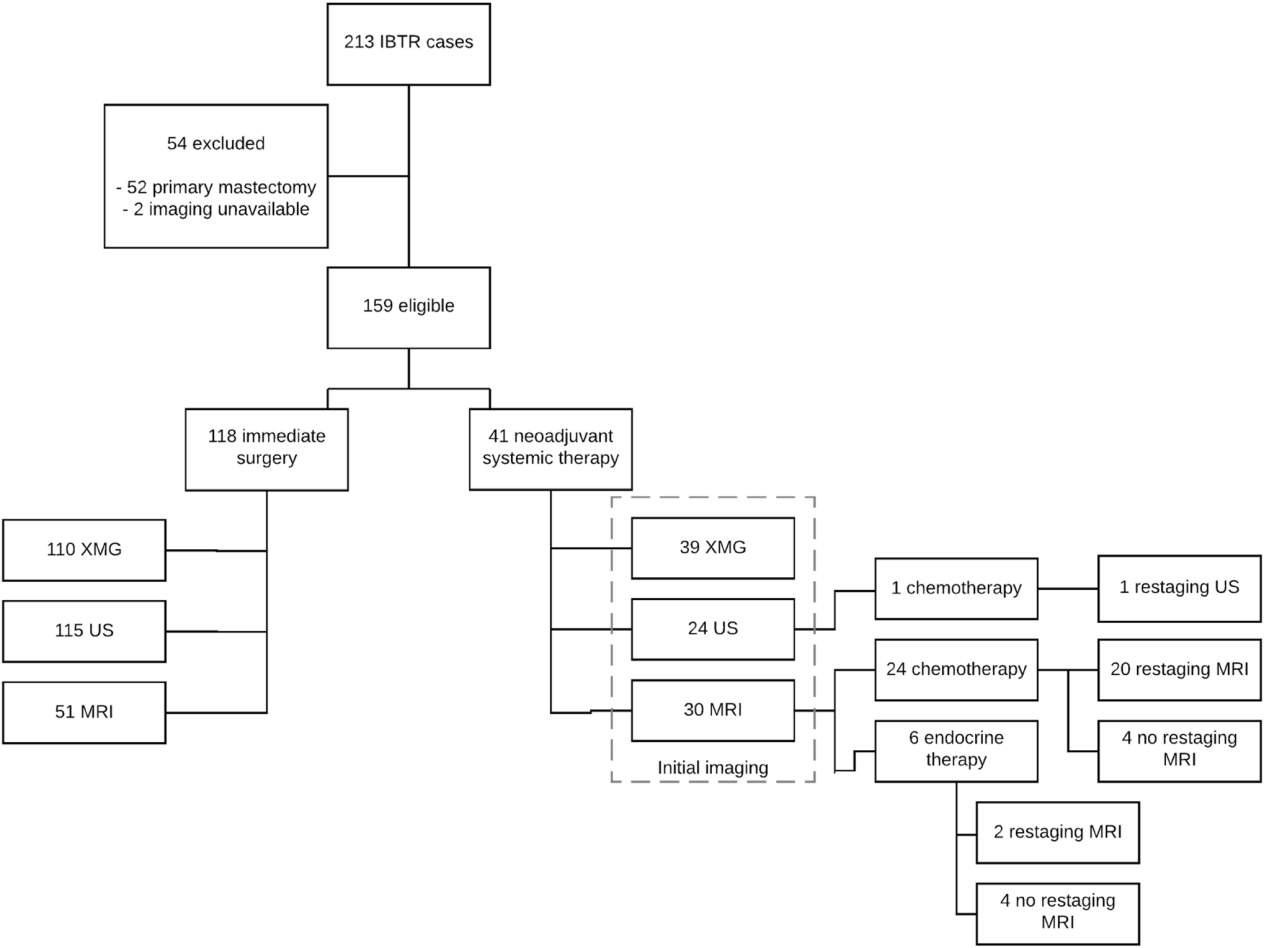
Flowchart of inclusion, treatment, and imaging techniques

Of the 213 screened patients with IBTR, 159 were eligible for inclusion (see Fig. 1). Of the IBTRs, 62.9% was screening detected and 37.1% self-reported. Of these 159 patients, 118 underwent surgery without prior systemic therapy, whereas 41 were treated with NST. In the immediate surgery group 110 XMGs, 115 USs, and 51 MRIs were available for analysis. Twenty-three cases (56%) in the NST group had evaluation imaging meeting our inclusion criteria: one by US and 22 by MRI.

Baseline characteristics of the included patients are presented in Table 1. The median age at diagnosis of IBTR was 62 years, with a median time to IBTR of 104 months. Almost all patients (91.4%) underwent radiotherapy as part of their primary BCT, whereas 20% had chemotherapy and 23% had endocrine therapy. Most common were recurrences with invasive ductal carcinoma (69.8%) and invasive lobular carcinoma (17.6%). Receptor statuses of all invasive recurrences were ER/PR+HER2− in 112/149 (75.2%), ER/PR−HER2+ in 4/149 (2.7%), ER/PR+HER2+ in 8/149 (5.4%), and ER/PR/HER2− in 22/149 (14.8%). The receptor statuses of patients with a recurrence of DCIS were not determined.

**Table 1:** Baseline characteristics (*N* = 159)

	All (*N* = 159)	Surgery (*N* = 118)	NST (*N* = 41)	*p* value
Median age at primary diagnosis (range)	51 (29–81)	52 (31–81)	48 (29–73)	0.142
Median time to IBTR in months (range)	104 (10–354)	105 (20–354)	94 (10–182)	0.137
Time to IBTR				
< 2 years	9 (5.7%)	6 (5.1%)	3 (7.3%)	0.594
> 2 years	150 (94.3%)	112 (94.9%)	38 (92.7%)	
Median age at IBTR diagnosis (range)	62 (34–88)	64 (34–88)	57 (34–82)	0.012*
Size IBTR at initial imaging (XMG, mm, range)	14.43 (12.14–16.72)	14.19 (11.72–16.65) (*N* = 110)	15.13 (9.60–20.65) (*N* = 39)	0.722
Adjuvant therapy primary tumor				
Radiotherapy	146 (91.8%)	106 (89.8%	40 (97.6%)	0.120
Chemotherapy	32 (20.0%)	26 (22.0%)	6 (14.6%)	0.309
Endocrine therapy	38 (23.9%)	27 (22.9%)	11 (26.8%)	0.650
Detection method IBTR				
Self-reported	59 (37.1%)	38 (32.2%)	21 (51.2%)	0.030*
Screening detected	100 (62.9%)	80 (67.8%)	20 (48.8%)	
Salvage surgery IBTR				
Mastectomy	149 (93.7%)	108 (91.5%)	41 (100%)	0.054
Repeat BCS	10 (6.3%)	10 (8.5%)	0 (0%)	
Contralateral mastectomy				
After imaging and biopsy malignant	7 (4.4%)	7 (5.9%)	0 (0%)	0.107
Patient’s request	3 (1.9%)	2 (1.7%)	1 (2.4%)	
IBTR tumor type				
DCIS	11 (6.9%)	10 (8.5%)	1 (2.4%)	0.579
IDC	111 (69.8%)	81 (68.6%)	30 (73.2%)	
ILC	28 (17.6%)	21 (17.8%)	7 (17.1%)	
Other	9 (5.7%)	6 (5.1%)	3 (7.3%)	
IBTR recurrence				
Unifocal (histopathology)	117 (73.6%)	88 (74.6%)	29 (70.7%)	0.346
Multifocal (histopathology)	35 (22%)	29 (24.6%)	6 (14.6%)	
Unknown	7 (4.4%)	1 (2.4%)	6 (14.6%)	
Receptor status				
ER/PR+, Her2Neu−	112 (70.4%)	83 (70.3%)	29 (70.7%)	0.590
ER/PR+, Her2Neu+	8 (5%)	7 (5.9%)	1 (2.4%)	
ER/PR−, Her2Neu−	22 (13.8%)	14 (11.9%)	8 (19.5%)	
ER/PR−, Her2Neu+	4 (2.5%)	3 (2.5%)	1 (2.4%)	
Unknown	13 (8.2%)	11 (9.3%)	2 (4.9%)	

*NST* Neoadjuvant systemic therapy, *IBTR* ipsilateral breast tumor recurrence, *XMG* mammography, *DCIS* ductal carcinoma in situ, *IDC* invasive ductal carcinoma, *ILC* invasive lobular carcinoma, *ER* estrogen receptor, *PR* progesterone receptor. *p* values marked with an asterisk are considered statistically significant (*p*  < 0.05)

Patients who underwent an MRI (76/159) in the workup of IBTR were significantly younger (mean 56.7 years vs. 64.2 years without MRI, *p* < 0.001). Tumor sizes on XMG were not significantly larger in the MRI group (*p* = 0.345 mean size on XMG in the XMG-only group vs. mean size on XMG in the XMG and MRI group). Furthermore, the size of IBTR found on the initial XMG did not differ significantly between the immediate surgery and NST group (*p* = 0.722, see Table 1).

### Size estimation

Tumors were occult (size = 0 mm) on 37/149 XMGs (24.8%), 14/148 USs (9.5%) ,and 3/81 MRIs (3.7%).

The mean differences between the size of IBTR measured by XMG, US, and MRI versus the size measured at histopathological examination were 3.48, 4.81, and 1.05 mm, respectively.

**Fig. 2:**
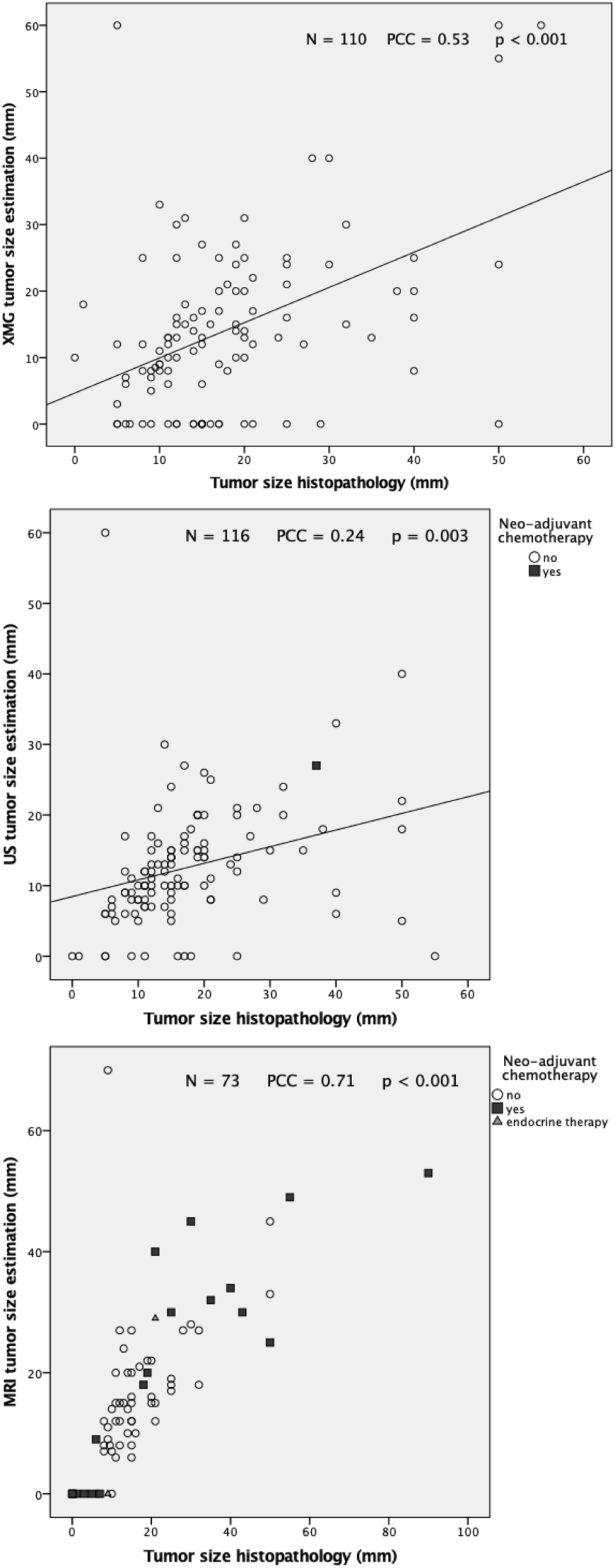
Scatter plots and Pearson’s correlation coefficients (PCC) for XMG, US, and MRI size estimation compared to histopathology findings, with statistical significance (*p* value). The outlier in the left upper corner of the XMG and US plot was a ductal carcinoma surrounded by DCIS which was largely overestimated on US and XMG (an MRI was not performed). The outlier in the left upper corner of the MRI plot was a lobular carcinoma which was overestimated on MRI, but adequately estimated by XMG and US

**Fig. 3:**
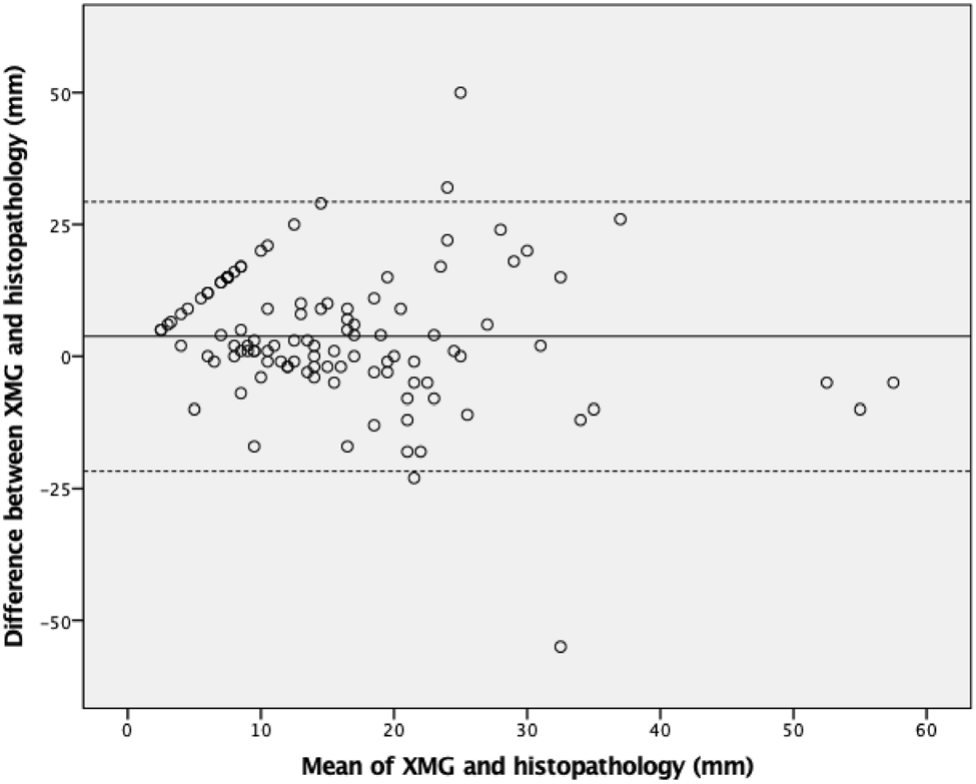
Bland–Altman plot showing size estimation accuracy for XMG. The continuous line represents the mean difference between XMG and histopathology (3.80 mm) and the dotted lines represent the limits of agreement (LOA) defined as the mean ± 1.96 times the standard deviation of the mean. All patients in this plot had surgery without NST (*N*  = 110)

**Fig. 4:**
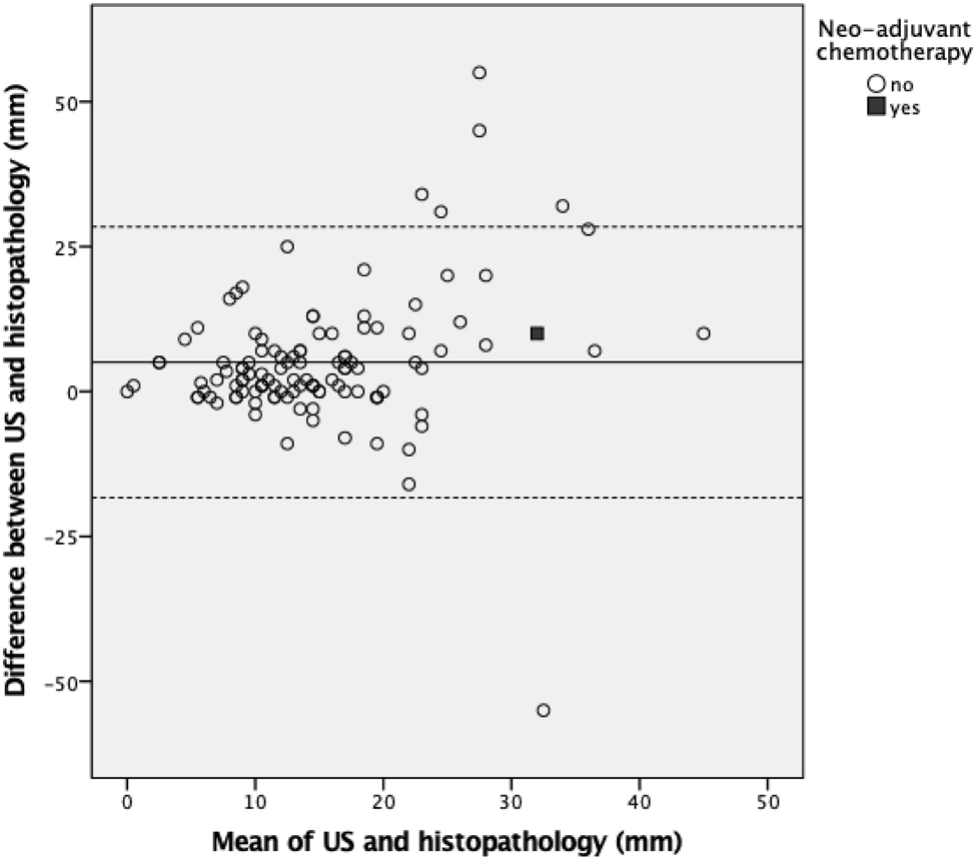
Bland–Altman plot showing size estimation accuracy for US (displayed by NST, *N*  = 116). The continuous line represents the mean difference between US and histopathology (5.04 mm) and the dotted lines represent the limits of agreement (LOA) defined as the mean ± 1.96 times the standard deviation of the mean

**Fig. 5:**
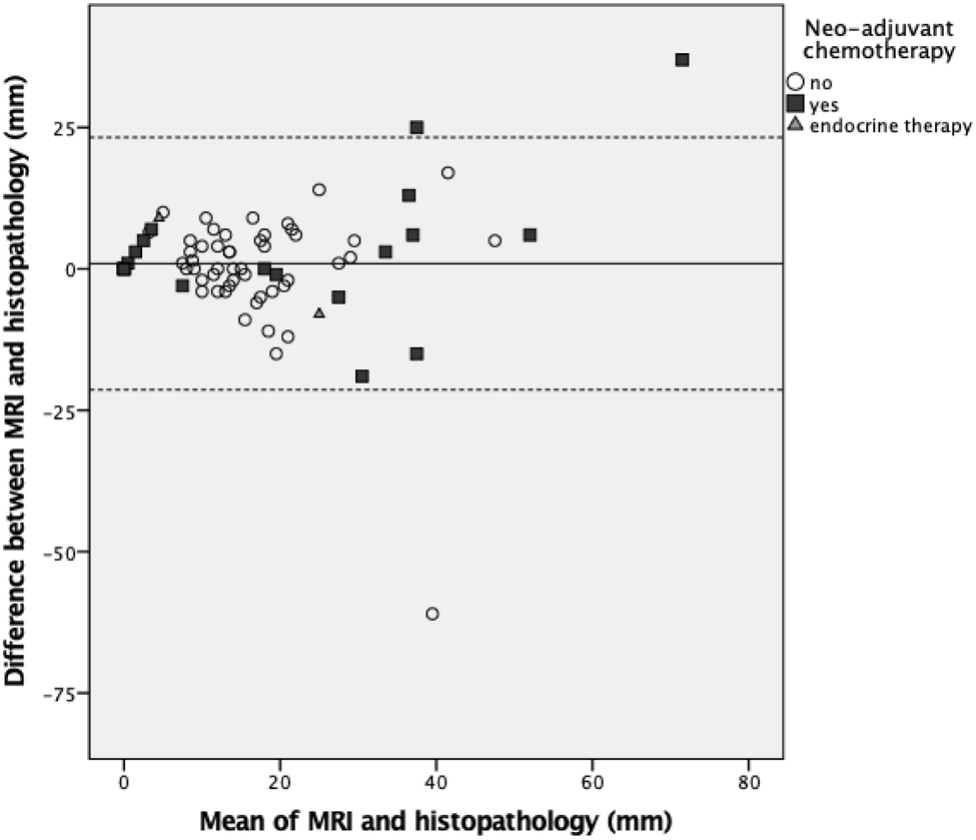
Bland–Altman plot showing size estimation accuracy for MRI (displayed by NST, *N*  = 73). The continuous line represents the mean difference between MRI and histopathology (1.05 mm) and the dotted lines represent the limits of agreement (LOA) defined as the mean ± 1.96 times the standard deviation of the mean

Both XMG and US showed a significant underestimation of the actual tumor size (*p* < 0.001 and *p* = 0.002, respectively, see Table 2), whereas MRI provided an adequate size estimation. The Pearson’s correlation coefficient (PCC) was significant for all three imaging techniques (see Table 2, Fig. 2). Figures 3, 4 and 5 shows Bland–Altman plots of XMG, US, and MRI size estimation agreement to the tumor size found in histopathology. The limits of agreement (LOAs) show that in 95% of cases the actual tumor size was between + 2.50 cm and − 2.12 cm of the XMG measurement, between + 2.8 cm and − 1.8 cm of the US measurement, and between + 2.23 cm and − 2.02 cm of the MRI measurement.

**Table 2:** Mean differences between size estimation and Pearson’s correlation coefficient

	Mean difference (mm)	95% CI (mm)	Pearson’s correlation coefficient (PCC)	p value PCC
Histopathology vs. XMG	3.48 (*p* = 0.003)	1.20 to 5.74	0.29	< 0.001
Histopathology vs. US	4.81 (*p* < 0.001)	2.68 to 6.93	0.21	0.011
Histopathology vs. MRI	1.05 (*p* = 0.416)	−1.52 to 3.63	0.71	< 0.001

The administration of NST had no statistically significant influence on the accuracy of size estimation by MRI: the mean difference between histopathology and MRI was 0.25 mm in the immediate surgery group vs. 2.91 mm on MRI after NST (*p* = 0.349, *N* = 73). Bland–Altman plots of these results can be found in supplement A.

### Multifocality

In the immediate surgery group (*N* = 118, 109 XMG and 51 MRI), ipsilateral additional lesions were detected by XMG in 5.5% (6 out of 109), and by MRI in 25.5% of patients (13 out of 51). Histopathology showed multifocality in 29/118 surgical specimens (24.6%). The additional value of MRI in the detection of multifocality could be assessed in 48 patients who had both XMG and MRI and did not undergo NST (see Tables 3 and 4). The sensitivity for multifocality in these 48 cases was significantly better for MRI (*p* = 0.008), whereas the specificity showed no significant difference (*p* = 0.307). The positive and negative predictive value (PPV and NPV) were not significantly different (*p* = 0.700 and *p* = 0.082, respectively) between XMG and MRI (Tables 3 and 4).

**Table 3:** Sensitivity, specificity, NPV (negative predicitve value), and PPV (positive predictive value) of multifocality detection by mammography (XMG) and histopathology (HP)

XMG	HP			
	Unifocal	Multifocal	Total	
Unifocal or occult	34	10	44	NPV 77.3%
Multifocal	1	3	4	PPV 25%
Total	35	13	48	
	Specificity 97.1%	Sensitivity 23.1%

**Table 4:** Sensitivity, specificity, NPV (negative predicitve value), and PPV (positive predicitve value) of multifocality detection by breast MRI and histopathology (HP)

MRI	HP			
	Unifocal	Multifocal	Total	
Unifocal or occult	32	3	35	NPV 91.4%
Multifocal	3	10	13	PPV 76.9%
Total	35	13	48	
	Specificity 91.4%	Sensitivity 76.9%		

### Contralateral findings

Imaging of the contralateral breast was performed in 151 patients (95%), with 143 XMGs, 15 USs, and 81 MRIs.

A contralateral lesion was detected in 14 patients (see Table 5). 7/14 proved to be malignant, 3/14 proved to be benign at biopsy, and 4/14 were not biopsied, but were regarded benign and proved to be benign at subsequent imaging. Of all 14 lesions, six were detected by XMG. Four of these were biopsied and proved to be malignant, leading to contralateral mastectomy. MRI detected eight lesions that were occult on XMG (in one case an XMG of the contralateral breast was not performed). Of these, six were biopsied; three were malignant and three benign. All three malignant biopsies led to a contralateral mastectomy. Two MRI-detected lesions were regarded benign without biopsy and proved to be benign at follow-up imaging.

**Table 5:** Detection, diagnostics, and surgical consequences of contralateral findings

Case no	XMG	MRI	Biopsy	Additional surgery
1	+	NP	Malignant	Contralateral mastectomy
2	+	+	Malignant	Contralateral mastectomy
3	+	+	Malignant	Contralateral mastectomy
4	+	+	Malignant	Contralateral mastectomy
5	+	−	NP	None
6	+	NP	NP	None
7	NP	+	Malignant	Contralateral mastectomy
8	−	+	Benign	None
9	−	+	Malignant	Contralateral mastectomy
10	−	+	Malignant	Contralateral mastectomy
11	−	+	Benign	None
12	−	+	Benign	None
13	−	+	NP	None
14	−	+	NP	None

+ , contralateral finding; − , no contralateral finding, *NP* not performed

Overall, the definitive contralateral malignancy finding rate in this cohort was 4.4% (8/159 patients, of which one was detected only after contralateral mastectomy at the patient's wish). Of these, 3 (37.5%, 1.9% of the cohort) would not have been detected without MRI.

## Discussion

This retrospective cohort study assessed the additional value of MRI in the preoperative workup for IBTR in the previously conservatively treated breast, regarding the measurement of the size of IBTR and the detection of multifocality and contralateral malignancies. XMG and US tended to underestimate IBTR tumor size, whereas MRI provided an accurate measurement. Furthermore, MRI had a significantly higher sensitivity for the detection of multifocality and contralateral malignancies.

To our knowledge, this is the first study to elaborate on this subject. It confirms that, like in primary breast cancer [6], MRI is the most accurate technique to measure the size of IBTR. With the recent trend towards repeat BCS for selected patients, MRI may provide a reliable measurement of the tumor size and detection of additional lesions, which allows for a precise selection of patients who could be eligible for repeat BCS.

During follow-up after BCT for primary breast cancer, the distinction between scar tissue and IBTR can be challenging. Surgery and radiotherapy cause architectural changes in the breast, which can be mistaken for, or mask, a tumor recurrence on XMG [32]. By contrast enhancement, MRI has shown to differentiate better between fibrosis and tumor recurrence [33]. This may affect the estimation of IBTR size, too, especially when the recurrence is at or near the lumpectomy site. However, the reliability of MRI is limited in the first year after primary BCT, due to post-radiation inflammatory findings [34]. This was unlikely to play a role in the current study population, as in 158/159 patients (99.3%) the IBTR occurred after more than 1 year after primary BCT.

The Pearson's coefficients of XMG and US suggested a weak to moderate correlation between the estimation of tumor size on imaging and histopathology, but were still significant. For MRI, the Pearson's coefficient suggested a strong correlation between imaging and histopathology. The Bland–Altman plots confirm the good agreement of all three imaging techniques with the tumor size later found in histopathology.

In this study, no clinically relevant difference was observed regarding size estimation using breast MRI in patients treated with primary surgery versus NST followed by surgery (see Supplement A). A recent meta-analysis by Marinovich et al. [37] suggested a slight overestimation of the actual tumor size after NST with pooled LOAs of − 4.2 and + 4.4 cm of the measured size on MRI. In contrast, our study showed a slight underestimation of tumor size on MRI after NST, with smaller LOA's of approximately − 2.3 cm and + 2.5 cm. This difference may be explained by the limited size of this study (*N* = 22 MRIs after NST) compared to the 953 patients in Marinovich's meta-analysis. Notwithstanding the limited sample size of this study, it suggests that breast MRI is the technique of preference for tumor size estimation in patients with IBTR, regardless of the use of NST.

When considering repeat BCS, a high sensitivity for the detection of multifocality in patients with IBTR is of vital importance. Local recurrence can be a sign of tumor aggressiveness, as can multifocality, and thus, the a priori probability of finding additional tumor foci in patients with IBTR is high. In this study, multifocality in the surgical specimen was present in 22% of cases. The risk of multifocality in patients with primary breast cancer is approximately 10% [12]. Whereas after primary BCS radiologically occult lesions could theoretically be eradicated by whole-breast radiotherapy, repeat whole-breast external beam radiotherapy is generally considered a contraindication in the previously irradiated breast due to the risk of skin toxicity. The feasibility of partial breast re-irradiation after a repeat BCS is currently under investigation and preliminary results are promising [38–41]. However, this technique does not allow the elimination of any occult lesions in other quadrants of the breast. The sensitivity for multifocality in this study was significantly higher for MRI when compared to XMG, which justifies the use of MRI when a repeat BCS is considered.

The reported sensitivity of MRI for the detection of ipsilateral additional lesions in primary breast cancer in literature varies from 78 to 100% with a specificity of 53–80% [42, 43]. In this study, the sensitivity of MRI was slightly lower than in primary cancer but still significantly higher than XMG (76.9% vs. 23.1%, *p* = 0.008), with a very high specificity (91.4%). We cannot rule out. A workup bias and thus an overestimation of the sensitivity and the specificity of MRI, as the suspicion of multifocality on XMG could have been a reason to perform an MRI. Another limitation of this retrospective study design is the relatively small number of MRIs performed, resulting in wide confidence intervals for the estimates of the sensitivity, specificity, and predictive values.

Due to the limited number of included patients, no sub-analyses for tumor type and grade were performed in this study. It would be interesting to compare the additional value of MRI for lobular and ductal IBTR, and for tumors with different tumor grade and hormone receptor and HER2 status. Furthermore, a sub-analysis using clonality testing ('true recurrence' or new primary tumor [44, 45]) could have added to the clinical significance of this study, as the prognosis for true recurrences seems to be worse than for new primary tumors [46, 47]. Nonetheless, this relatively small cohort study is the first to investigate the additional value of MRI in IBTR. These findings need to be evaluated in larger groups to allow for sub-analyses.

Regarding the presence of metachronous contralateral breast cancer, there appears to be an oncological benefit of early detection [48, 49]. The incidental finding of a contralateral malignancy in this population (4.4%) is in concordance with literature [50]. MRI detected three contralateral malignancies that were occult on XMG (1.9% of all patients). In previous studies, MRI as part of follow-up after BCT detected a metachronous contralateral tumor in approximately 4% of patients [17].

## Conclusion

The addition of breast MRI to XMG and US in the preoperative [8, 9] workup of IBTR allows for a more accurate assessment of the size of IBTR and provides a higher sensitivity for multifocality in the ipsilateral breast. When considering repeat breast-conserving surgery, MRI is a reliable tool for surgical planning. The added value of MRI in the detection of contralateral tumors seems limited.

## Electronic supplementary material

Below is the link to the electronic supplementary material.

Supplementary file1 (DOCX 57 kb)
