# Supplementary material for: Local staging of ipsilateral breast tumor recurrence: mammography, ultrasound, or MRI?
Source: Breast Cancer Res Treat. 2020 Aug 8;184(2):385–95. doi: 10.1007/s10549-020-05850-9 (PMC7599170; doi:10.1007/s10549-020-05850-9)
Supplement: Supplementary file 1 — Supplementary file1 (DOCX 57 kb) [file 10549_2020_5850_MOESM1_ESM.docx]

**SUPPLEMENT A**

|  | Mean difference (mm) | 95%-CI (mm) | p-value* | Pearson’s correlation coefficient (PCC) | p-value PCC |
| --- | --- | --- | --- | --- | --- |
| Without NST | 0.08 | -2.75 - 2.92 |  | 0.53 | < 0.001 |
| After NST | 3.59 | -1.32 – 8.51 | 0.118 | 0.87 | <0.001 |

**Supplement A1** - Comparison of mean differences between size estimation on MRI and histopathology for patients with and without neoadjuvant systemic therapy (NST). *Comparison of the mean differences between the groups (student’s T-test)


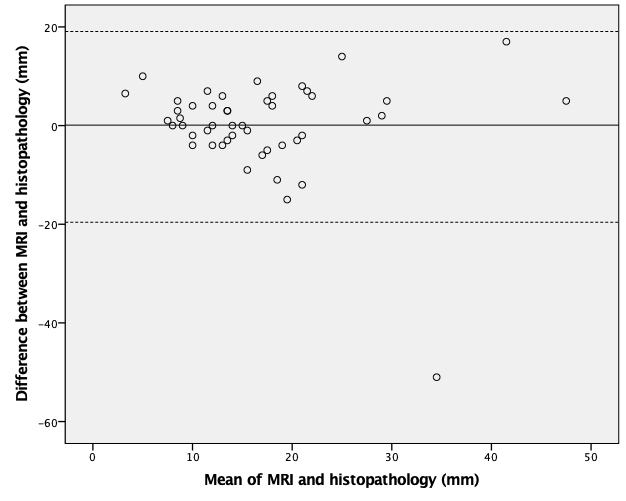


**Supplement A2** - Bland-Altman plot of MRI size estimation without neoadjuvant systemic therapy (N = 51)


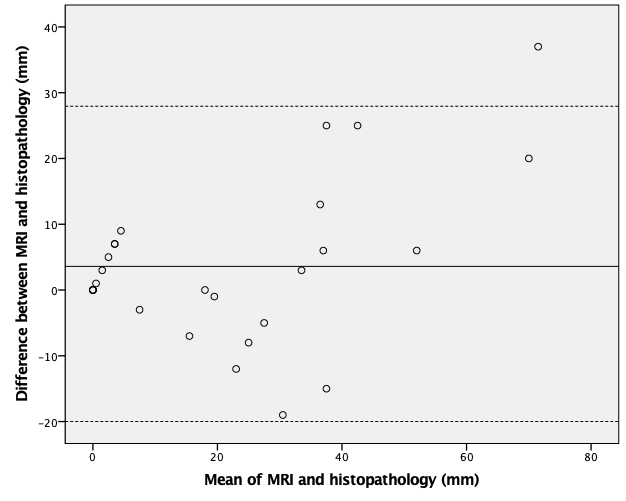


**Supplement A3** - Bland-Altman plot of MRI size estimation after neoadjuvant systemic therapy (N = 22)
